# Online Correction Method for the Registration Error between TSMFTIS Detector and Interferogram

**DOI:** 10.3390/s20041195

**Published:** 2020-02-21

**Authors:** Jun Cao, Yan Yuan, Lijuan Su, Conghui Zhu, Qiangqiang Yan

**Affiliations:** 1 Key Laboratory of Precision Opto-mechatronics Technology Sponsored by Ministry of Education, School of Instrumentation Science and Opto-Electronics Engineering, Beihang University, Beijing 100191, China; 2 Key Laboratory of Spectral Imaging Technology of Chinese Academy of Sciences, Xi’an 710119, China

**Keywords:** temporally–spatially-modulated Fourier transform imaging spectrometers (TSMFTISs), robust least-square (RLS) linear fitting, registration error, interferogram

## Abstract

Temporally-spatially modulated Fourier transform imaging spectrometers (TSMFTISs) provide high-throughout-type push-broom spectrometry with both temporal and spatial modulation features. The system requires strict registration between the detector and the interferogram. However, registration errors are unavoidable and directly change the corresponding optical path difference values of the interferogram. As a result, the interferogram should be corrected before restoring the spectrum. In order to obtain the correct optical path difference (OPD) values, an online registration error correction method based on robust least-square linear fitting is presented. The model of the registration error was constructed to analyze its effect on the reconstructed spectra. Fitting methods were used to obtain correct optical path difference information. Simulations based on the proposed method were performed to determine the influence of the registration error on the restored spectra and the effectiveness of the proposed correction method. The simulation results prove that the accuracy of the recovered spectrum can be improved after correcting the interferogram deviation caused by the registration error. The experimental data were also corrected using the proposed methods.

## 1. Introduction

Hyperspectral imaging is an important technology used for obtaining spatial and spectral information of an object of interest. It has been widely applied in many areas, for instance, prospecting and soil composition determination in geology [1,2], assessing water contamination in hydrology [3], identifying different crop fields and monitor water in agriculture [4], vegetation classification in biology [5], and intelligence acquisition in the military arena [6]. Temporally–spatially-modulated Fourier transform imaging spectrometers (TSMFTIS) are a type of push-broom hyperspectral imaging sensor based on the principle of lateral shearing interferometer [7]. The instantaneous field of view of TSMFTIS is two-dimensional; one pixel of the scene is associated to only one pixel of the detector, and all the available optical path differences (OPDs) of each pixel of the scene are obtained by a perpendicular and full field push-broom over the scene. The spectrum of the scene points can be recovered from the rebuilt interferogram. Thus, the TSMFTISs obtain three-dimensional information (two-dimensional spatial information and one-dimensional spectrum information). TSMFTISs were developed as airborne/spaceborne hyperspectral imagers for earth remote sensing because of its high throughput advantage. Over a long period, the TSMFTISs developed include the large aperture static imaging spectrometer (LASIS) [8,9], the high etendue imaging Fourier transform spectrometer (HEIFTS) [10,11], Caméra Hyperspectrale de Démonstration (CaHyD) [12], the aerospace leap-frog imaging static interferometer for Earth observation (ALISEO) [13,14], the medium- and long-wave infrared spectral imaging instrument SIELETERS [15,16], and so on.

The data obtained by TSMFTIS are interference datacubic and need to go through further transformations before use. Because of the influence of various errors, the data processing of TSMFTIS is more complicated. In practice, registration errors occur between the spatial direction of the image of its imaging system and the pixel column of its detector. As a result, the OPDs correspond to the interferogram changes, resulting in an inability to accurately recover the spectral information. Zhang et al. [8] constructed a correction system with two theodolites, rotating table and parallel optical tubes, calibrated the detector registration error, calculated the centroid position of the star at the position of zero OPD of the spectral dimension, and obtained the registration error by fitting the slope of the line with the least-squares line. The calibration method has complex operation, high computational complexity, and no real-time ability. To our best knowledge, no study reported an online real-time correction method for the error between the detector and interferogram of TSMFTIS.

In this paper, we introduce an online correction method for the registration error between the detector and interferogram of TSMFTISs based on the robust least-squares method. Online means that the correction method can calculate the registration error in real time from the obtained image of the TSMFTIS without any other added measuring data and correct the registration error with high accuracy and robustness. The paper is organized as follows: In Section 2, we briefly review the principles of TSMFTISs and the theory of Fourier transform spectroscopy. In Section 3, the model of the registration error between the detector and the interferogram of TSMFTIS is established, and the characteristics of the influence of the registration error on imaging are analyzed. In Section 4, the proposed correction method is introduced. In Section 5, the simulation demonstrates the effectiveness of the proposed method. Then, the proposed method is implemented on actual interference-modulated images. Finally, we draw our conclusions.

## 2. Principles of TSMFTIS

**Figure 1:**
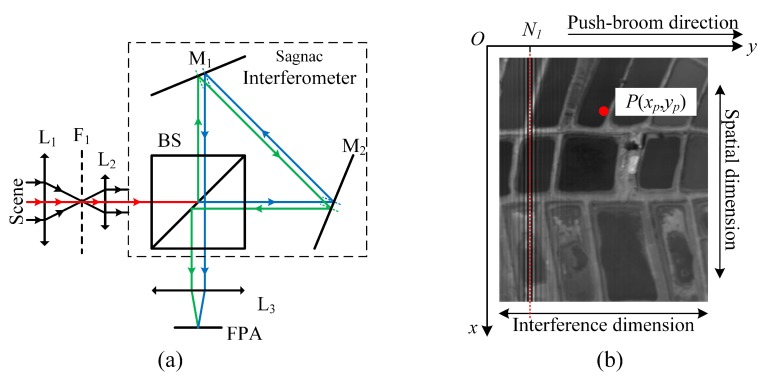
(**a**) Temporally–spatially-modulated Fourier transform imaging spectrometer (TSMFTIS) system and (**b**) the interference image obtained by TSMFTIS.

Figure 1a illustrates the optical layout of a TSMFTIS system [17], which includes fore-optics $L_{1}$, a collimator $L_{2}$, a Sagnac interferometer, a Fourier lens $L_{3}$, and a focal plane array (FPA) detector. The incident light from the scene is first collimated by the lens $L_{1}$ and $L_{2}$, and travels the interferometer by means of a beam-splitter BS and two folding mirrors $M_{1}$ and $M_{2}$. The interferometer forms two virtual images of the scene that match each other geometrically. Light emerging from the output port of interferometer is then focused onto the FPA by the lens $L_{3}$. Due to the existence of lateral shearing, the optical path lengths of the two virtual images are different, so the image of the scene obtained by the instrument is modulated with stationary interference fringes. A typical TSMFTIS image is shown in Figure 1b. The image can be seen as a common image of the scene modulated with a stationary interference pattern. The common image value can be seen as the (direct-current) DC component, and the pattern can be seen as the fluctuant component. When the fluctuant component is much smaller than the DC component, the pattern is not very obvious. This leads to the interference pattern, which seems to only be present in some image regions. Ideally, the pixels in a column of the detector should correspond to the same OPD, and the direction of the column is noted in the spatial dimension. The pixels in a row have different OPDs, and the direction of the row is noted in the interference direction. When the instrument is pushed through a scene in the direction vertical to the column, a target in the scene is captured in a series of images by pixels in a row that have different OPDs.

A coordinate is defined as shown in Figure 1b. The x-axis represents the spatial dimension and the y-axis represents the interference dimension. Ideally, the interference intensity at position *P* can be expressed as:

(1) $$I\left(x_{p},y_{p}\right)=\int_{v_{1}}^{v_{2}}B\left(v\right)\exp\left(2\pi v\frac{d}{f}\left(y_{p}-N_{1}\right)j\right)dv$$

where *d* denotes the lateral shearing of the interferometer, *f* denotes the focus of the Fourier lens, ($x_{p}$, $y_{p}$) denotes the spot position of the object *P* on FPA, $N_{1}$ denotes the column axis of the zero OPD position, *j* represents an imaginary unit, *v* represents the wavenumber, and *B*(*v*) is the spectral radiance of the target corresponding to polychromatic light the spectral range from $v_{1}$ to $v_{2}$.

**Figure 2:**
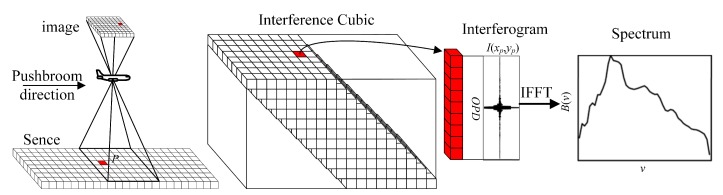
Schematic of TSMFTIS data collection and processing.

Figure 2 shows the data collection and processing workflow of a TSMFTIS. According to principles of TSMFTIS, the different image points on the FPA correspond to the different object points on the virtual surface with a certain OPD. When the TSMFTIS pushes through the scene to obtain the complete interference data of the targets, the target interference *I* can be obtained by extracting the intensity of interference sequentially from images as shown in Figure 2. Based on Fourier transform theory, the spectrum of the target can be reconstructed by inverse Fourier transform as follows:

(2) $$B\left(v\right)=\int_{0}^{L}I\left(x,y\right)\exp\left[-2\pi v\frac{d}{f}\left(y-N_{1}\right)j\right]dy$$

where (*x*,*y*) represents the position of the imaging point on the FPA and *L* denotes the distance to the zero OPD position, which corresponds to the maximum OPD position.

## 3. Modeling and Analysis of the Registration Error

**Figure 3:**
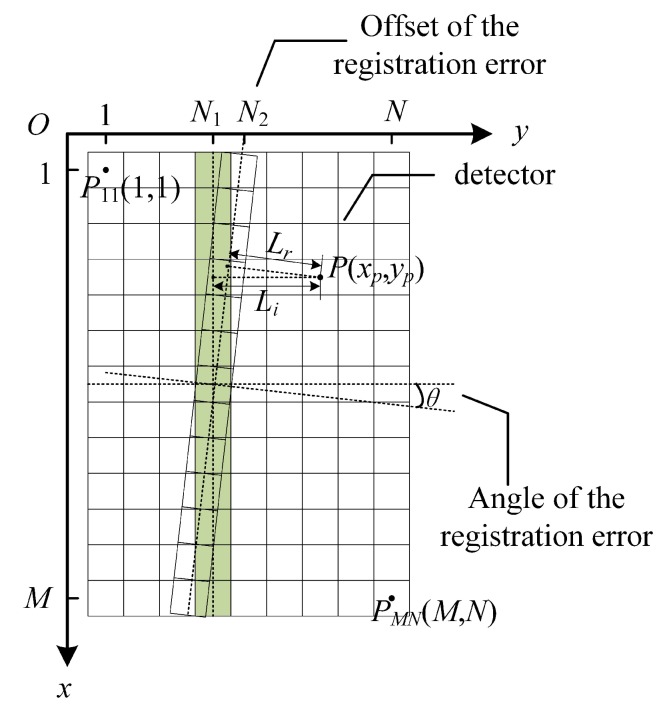
Schematic diagram of the registration error between the detector and the interferogram.

During manufacturing of the instrument, misalignment unavoidably occurs between the detector and the designed interferogram line. Based on the imaging principle of TSMFTIS, the OPD of each point on the interference image is a fixed value. For an ideal system, the positions corresponding to zero OPD should be located in a straight line of pixels. The misalignment between the detector and the interferogram destroys the desired one-to-one correspondence between the OPD positions of the ground object and the pixel positions. However, the zero OPD positions of the interferogram image formed by the imaging optics are still a straight line, as shown in Figure 3. In the figure, the registration error between the detector and the interferogram is associated with tilting angle θ and offset $N_{2}$. θ represents the angle between the pixel column of the detector array and the zero OPD line of the interferograms. $N_{2}$ represents the offset in the interference dimension.

Assume that $L_{i}$ denotes the ideal distance between the target point *P* and the zero OPD, and $L_{r}$ denotes the actual distance between the target point P and the zero OPD due to the existence of registration error. The OPD deviation of *P* is:

(3) $$\Delta OPD=\frac{d}{f}\left(L_{r}-L_{i}\right).$$

The ideal interference intensity of the target point *P* is expressed as:

(4) $$I\left(x_{p},y_{p}\right)=\int_{u_{1}}^{v_{2}}B\left(v\right)\exp\left(2\pi v\frac{d}{f}L_{i}j\right)dv.$$

The actual interference intensity of the target point *P* is:

(5) $$I^{\prime }\left(x_{p},y_{p}\right)=\int_{v_{1}}^{v_{2}}B\left(v\right)\exp\left(2\pi v\frac{d}{f}L_{r}j\right)dv.$$

**Figure 4:**
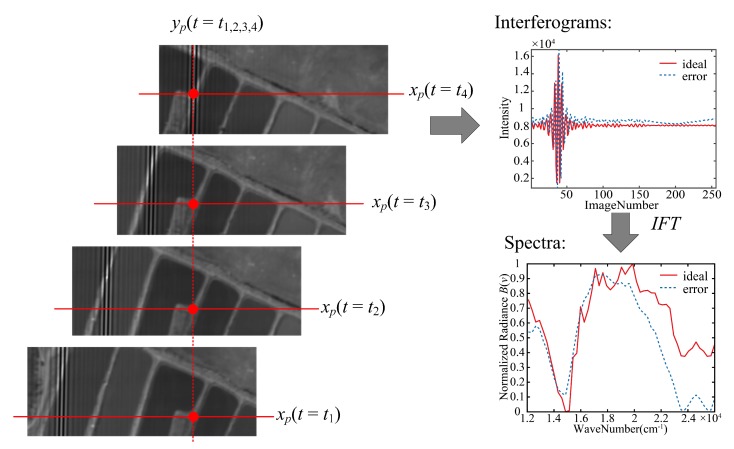
The influence of registration error on the interferogram and the recovered spectrum.

Comparing Equations (4) and (5), the interference intensity of each position in the interferogram output by the detector is changed. If the interferogram of the target is extracted, and using the ideal OPD value to reconstruct the spectrum, the recovered spectrum deviates from the real spectrum of target, as shown in Figure 4. In practice, the tilting, angle of the misalignment error between the detector and the interferogram line of our instrument is approximately 0.82${}^{\circ }$, which has a non-neglected negative impact on the recovery spectrum. Therefore, it is necessary to correct the OPD values of the raw data to eliminate the unfavorable effects caused by the registration error.

To model and calculate the registration error, the coordinates of the upper-left corner pixel of the image are set to (1,1) as shown in Figure 3. The size of the detector is M×N. Therefore, the equation of the straight line obtained by the ideal zero OPD positions located in the image is:

(6) $$y_{i}=N_{1}$$

Due to the misregistration, the equation of the straight line obtained by the actual zero OPD positions can be expressed as:

(7) $$y_{r}=\tan\theta \cdot x+N_{2}$$

where *x* and *y* denote the positions of zero OPD in the coordinate, tanθ denotes the slope of the line, and the $N_{2}$ denotes the Y-intercept. For the target point *P*($x_{p}$, $y_{p}$), the distant $L_{i}$ and $L_{r}$ can be calculated as follows:

(8) $$\left\{\begin{array}{c} L_{i}=\left|y_{p}-N_{1}\right|\\ L_{r}=\frac{\left|\tan\theta \cdot x_{p}-y_{p}+N_{2}\right|}{\sqrt{\tan^{2}\theta +1}} \end{array}\right.$$

According to Equations (3) and (8), the OPD of the target is only related to the value of $y_{p}$ in the ideal case; when misregistration occurs, the value of $x_{p}$ also affects the OPD. Since $\tan^{2}\theta \ll 1$ in this case, the OPD deviation of *P* can be rewritten and simplified as:

(9) $$\Delta OPD\approx \frac{d}{f}\left(\left|\tan\theta \cdot x_{p}-y_{p}+N_{2}\right|-\left|y_{p}-N_{1}\right|\right)$$

From Equation (9), when the TSMFTIS sweeps the entire field of view along the interference dimension strictly, the deviation of the OPD is almost a nonzero fixed value all the time. When the TSMFTIS sweeps the entire field of view nonstationarily, the deviation of OPD of the target point in the multiframe push-broom image sequence changes nonlinearly, and the distortion of the interferogram is serious. Therefore, the registration error must be corrected to match the OPDs and the intensities of interferogram in the data processing of TSMFTIS.

## 4. Proposed Correction Method

Since the interferograms of the target on both sides of the zero OPD location are exactly symmetrical, the interference information of the area with a uniform feature in the image is considered to be symmetric near the zero OPD. The position of the zero OPD is the extreme position of the interference curve. The extreme of each line appears as a straight and much brighter line in the image. Therefore, we propose a two-step correction method for the correction of the registration error between the detector and the interferogram. The first step employs the parabolic fitting method to find the zero OPD positions and their interference values. The second step uses these zero OPD positions to solve the registration error between the detector and the interferogram by employing linear fitting methods.

### 4.1. Parabolic Fitting Method for the Zero OPD Position

The parabolic fitting method is a mature mathematical tool. The parabola equation in this case can be expressed as:

(10) $$I=ay^{2}+by+c$$

When the coordinates *y* of three points in the line are known, the coefficients *a*, *b*, and *c* are obtained by solving the system of ternary linear equations. The independent variable *y* denotes the column position of the points, and the dependent variable *I* denotes the interference intensity value of target at position *y*. The y-coordinate position of the parabola’s vertex is calculated as:

(11) $$y_{m}=\frac{b}{-2a}$$

where $y_{m}$ is the position of the zero OPD column of the current row *m*, which is generally a non-integer value here.

We propose a method to automatically select the three points for parabolic fitting. The position of the ideal zero OPD is assumed to be the $N_{1}$ column on the detector. For the data in line *m*, a certain number of pixels before and after $N_{1}$ are employed. The information of these pixels can be expressed as ($y_{n}$,*I*(*m*,$y_{n}$)), where $y_{n}=N_{1}+n$ and *n* = 0, 1, 2, …, $N_{n}$. In this paper, $N_{n}$ = 8 is employed. Then, the pixel with the largest grey value among these integer points is selected, and its position is noted as $y_{k}$. The pixels before and after this pixel are selected. These three pixels are denoted as ($y_{k}-1$, *I*(*m*, $y_{k}-1$)), ($y_{k}$, *I*(*m*, $y_{k}$)), and ($y_{k}+1$, *I*(*m*, $y_{k}+1$)). Then, we put them into the parabolic Equation (10) and solve the parameters *a*, *b*, and *c*. According to Equation (11), we obtain a dataset of the zero OPD positions, which can be denoted as *m*, $y_{m}$, *m* = 1, 2, …, *M* for linear fitting.

### 4.2. Linear Fitting Method for Registration Error

The linear fitting method is also a mature mathematical tool. The equation of the line to be fitted in this case can be expressed as:

(12) $$y_{m}=km+t$$

where *k* is the slope of the line, *t* is the y-intercept, and *k* and *t* are undetermined parameters. The least-squares (LS) [18], total-least-squares (TLS) [19], and robust-least-squares (RLS) [20,21] linear fitting methods are three well- developed fitting methods. the LS method is the most commonly used. As errors might exist in the position coordinates of the zero OPD, the TLS method can be used to solve this type of linear fitting problem. If the zero OPD of one row in the image is located at the junction of different targets, the position of the zero OPD calculated by the parabola method is coarse and must be an outlier. These rough errors or outliers in the data need to be eliminated to obtain the optimal estimation of robust line parameters. The RLS method can be used in this kind of situation. The theory of the above methods is outlined below.

#### 4.2.1. Least Square Linear Fitting

The least-squares criterion of linear fitting is:

(13) $$e^{2}=\sum_{m=1}^{M}{∥km+t-y_{m}∥}^{2}=\min.$$

Convert to the matrix form:

(14) $$e^{2}={∥PZ-L∥}_{2}^{2}=\min,$$

where $P={\left[\begin{array}{cccc} 1 & 2 & \cdots & M\\ 1 & 1 & \cdots & 1 \end{array}\right]}^{T},Z={\left[\begin{array}{cc} k & t \end{array}\right]}^{T},L={\left[\begin{array}{cccc} y_{1} & y_{2} & \cdots & y_{M} \end{array}\right]}^{T}$.

Calculate the least-squares solutions of the coefficients *k* and *t*:

(15) $$Z_{LS}={\left(P^{T}P\right)}^{-1}P^{T}L.$$

#### 4.2.2. Total-Least-Squares Linear Fitting

The equation of the TLS method is expressed as:

(16) $$y_{m}+e_{y_{m}}=k\left(m+e_{m}\right)+t$$

where $e_{m}$ and $e_{y_{m}}$ are error terms. The total-least-squares criterion of linear fitting is:

(17) $$e_{m}^{2}+e_{y_{m}}^{2}=\min.$$

To solve this problem, construct the augmented matrix *C*:

(18) C=[P,L]

and conduct singular value decomposition for the augmented matrix *C*:

(19) $$C=USV^{T}$$

where $V^{T}=\left[\begin{array}{cc} V_{11} & V_{12}\\ V_{21} & V_{22} \end{array}\right]$, $V_{22}$ is scalar. The total-least-squares solution of the parameter is:

(20) $$Z_{TLS}=-V_{12}V_{22}.$$

#### 4.2.3. Robust-Least-Squares Linear Fitting

The specific process of the robust-least-squares method is as follows:

(1) Calculate the initial values of *k* and *t* by applying the LS method using all points;

(2) Calculate the distance $d_{m}$ from each point to the fitted line using the calculated *k* and *t* values:

(21) $$d_{m}=\frac{\left|km-y_{m}+t\right|}{\sqrt{k^{2}+1}};$$

(3) Set the threshold value of the abnormal point as *T*. If $d_{m}$ > *T*; this point is considered an abnormal point and eliminated; otherwise, it is reserved;

(4) Recalculate *k* and *t* using all reserved coordinate points;

(5) Repeat steps (2) to (4) until all coordinate points meet the requirements; and

(6) For the remaining points, the LS method is used to calculate the coefficients *k* and *t*, which can be seen as the optimal value.

In the above steps, LS can be replaced with TLS, which is another method called the robust-total-least-squares (RTLS) method.

In this paper, the threshold in step (3) is the responsibility of the RLS method. An optimal threshold *T* = 3σ is used in this paper, which was determined by trial and error. Here, σ is the standard deviation of the distance *d*:

(22) $$\sigma =\sqrt{\frac{{\left(d_{m}-\bar{d}\right)}^{2}}{M_{1}-1}}$$

where $\bar{d}=\frac{1}{M_{1}}\sum_{m=1}^{M_{1}}d_{m}$, $M_{1}$ is the number of points used for the linear fitting each time.

Once the linear equation established by the coefficients *k* and *t*, the registration error of each position between the detector and the interferogram can be calculated as follows:

(23) $$\left\{\begin{array}{c} \theta =\arctan k\\ N_{2}=t. \end{array}\right.$$

## 5. Simulation Results

**Figure 5:**
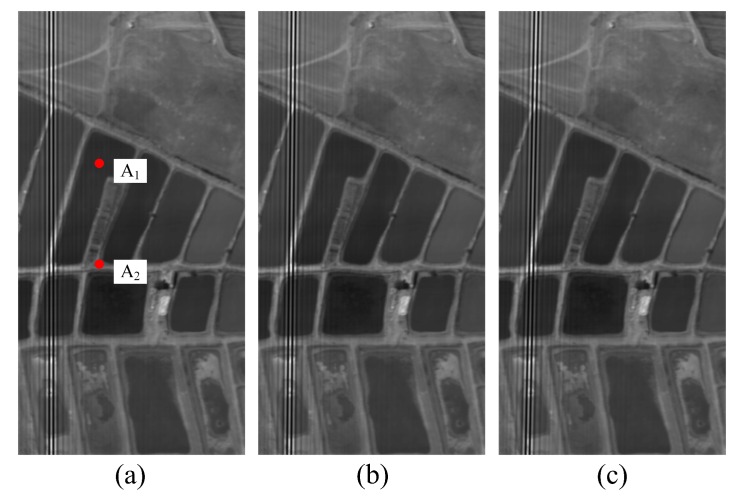
Simulation interference images with different registration errors between detector and interferogram: (**a**) *k* = 0, *t* = 38; (**b**) *k* = −0.01, *t* = 40.5; and (**c**) *k* = −0.02, *t* = 43.

An airborne hyperspectral scanner data set was employed to model the interferogram generated by a TSMFTIS. We assumed that the spectrum corresponds to 51 spectral bands within 13,405–22,222 cm${}^{-1}$. The equivalent focal length of the TSMFTIS is $f_{1}$ = 150 mm, and the shear constant of the TSMFTIS is $a_{1}$ = 0.84 mm. The FPA size is 256 × 500 pixels, and the pixel size *s* = 30 μm. The interference images generated with different registration errors are shown in Figure 5. To increase the clarity of the images, the contrast and brightness of the images were enhanced by grey expanding.

Four linear fitting methods, LS, TLS, RLS, and RTLS, were applied to the simulation images to calculate the registration error between the detector and the interferogram. The results of parameters of the fitting line are shown in Table 1.

**Figure 6:**
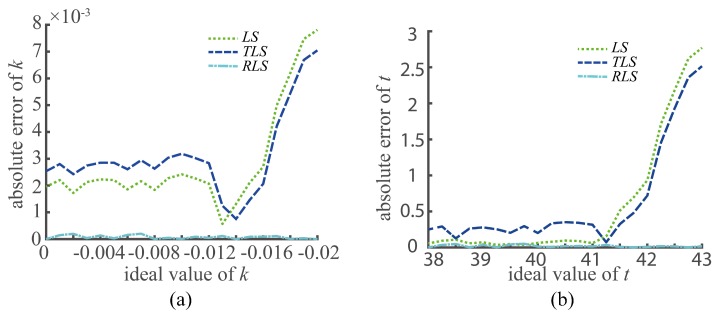
The error estimation of the fitting methods: (**a**) error of *k*; (**b**) error of *t*.

The results in the table show that the accuracy of the TLS method is the worst, followed by that of the LS method. The RLS and RTLS linear fitting methods have the same accuracy, and they are the closest to the value of preset. Since the operation speed of the RLS method is 10 times faster than that of the RTLS method in the actual calculation process, the RTLS method is not displayed in the rest of the paper. To estimate the error of the fitting methods, we calculated the absolute error between the ideal value and the fitting value of *k*,*t*, as shown in Figure 6. The figure shows that the RLS method produced robust and accurate results. Therefore, the RLS method was used as the optimal linear fitting method in this study.

The error was also quantitatively evaluated from the perspective of the recovery spectrum. The spectral angle (SA) [22,23] was employed to quantitative evaluate the spectral error of the reconstructed spectrum. The formula is as follows:

(24) $$SA\left(B,B^{\prime }\right)=\cos^{-1}\left(\sum_{i=1}^{N_{B}}B_{i}B_{i}^{\prime }/\left(\sqrt{\sum_{i=1}^{N_{B}}B_{i}^{2}}\sqrt{\sum_{i=1}^{N_{B}}B_{i}^{\prime 2}}\right)\right)$$

where *B* is the ideal spectrum radiance, $B^{\prime }$ is the reconstructed spectrum radiance, and $N_{B}$ is the number of spectrum channels.

**Figure 7:**
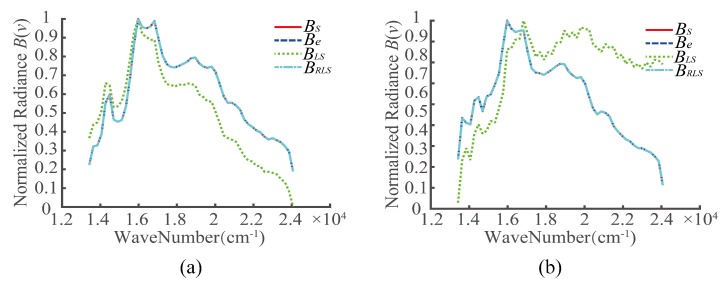
Recovery spectra of targets in case 1 (*k* = 0, *t* = 38): (**a**) $A_{1}$ and (**b**) $A_{2}$.

**Figure 8:**
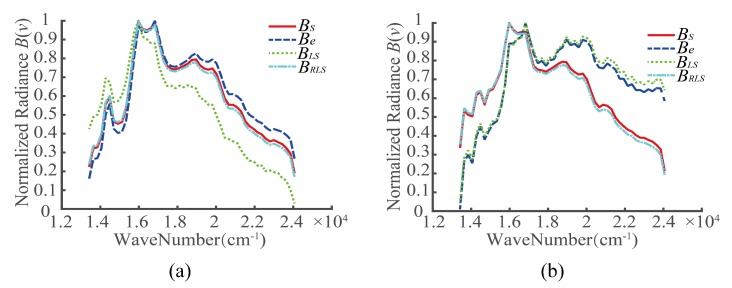
Recovery spectra of targets in case 2 (*k* = −0.01, *t* = 40.5): (**a**) $A_{1}$ and (**b**) $A_{2}$.

**Figure 9:**
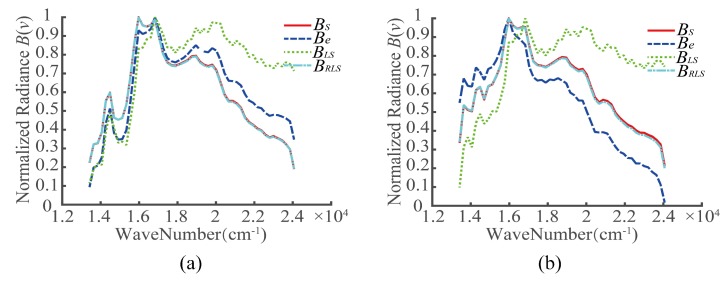
Recovery spectra of targets in case 3 (*k* = −0.02, *t* = 43): (**a**) $A_{1}$ and (**b**) $A_{2}$.

Two targets were selected, denoted by $A_{1}$ and $A_{2}$, as shown in Figure 5. For the registration errors of different detectors, the interferograms of $A_{1}$ and $A_{2}$ before the registration error correction, after the LS correction, and after the RLS correction were extracted. Then, the recovery spectra were obtained based on the Fourier spectroscopy principle, as shown in Figure 7, Figure 8 and Figure 9. In the figures, $B_{S}$ represents the ideal spectral curve of the target, $B_{e}$ represents the directly recovered spectral curve, $B_{LS}$ represents the recovered spectral curve after the registration error was corrected based on the LS method, and $B_{RLS}$ represents the recovered spectral curve after the registration error was corrected based on the RLS method.

The spectral angle of the recovered spectrum relative to the real spectrum was calculated, and the results are summarized in Table 2. In the table, Error denotes the SA between the real spectrum of the targets and the directly recovered spectrum based on the wrong OPD $L_{i}$. LS and RLS denote the SA between the real spectrum of the targets and the recovery spectra based on the OPD $L_{r}$ corrected by the LS method and the RLS method, respectively. Table 2 shows a large error existed between the real spectrum and the directly recovery spectrum when the registration errors. However, the LS method cannot effectively correct the registration error. The recovered spectrum obtained by the RLS method is much closer to the real spectrum.

## 6. Experimental Results

Next, we applied the proposed correction method to the interference-modulated image datasets obtained from an airborne TSMFTIS experiment to verify the effectiveness of this method. The TSMFTIS was mounted on an unstabilized airborne platform and collected 60 spectral bands within 900–2500 nm. The equivalent focal length of the TSMFTIS $f_{1}$ was 130 mm, and the shear constant of the TSMFTIS $a_{1}$ was 0.84 mm. The FPA size was 256 × 500 pixels, and the pixel size $p_{1}$ was 30 μm.

**Figure 10:**
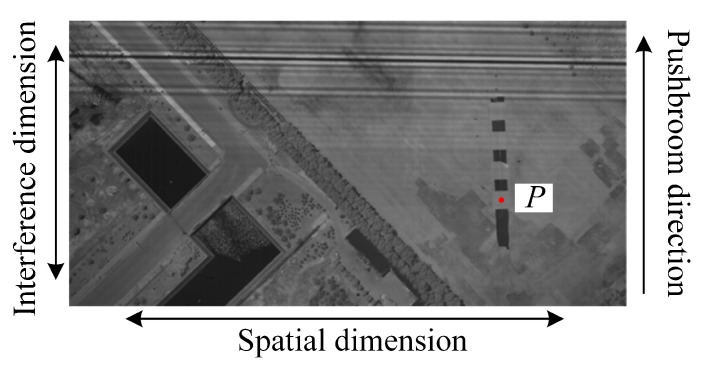
An experiment frame of an image obtained by the TSMFTIS.

A frame of an experimental image obtained by the airborne TSMFTIS is shown in Figure 10. A registration error is visible between the detector and the interference pattern. The LS and RLS methods were used to calculate the parameters of the fitted line for the registration error, and the results are shown in Table 3.

**Figure 11:**
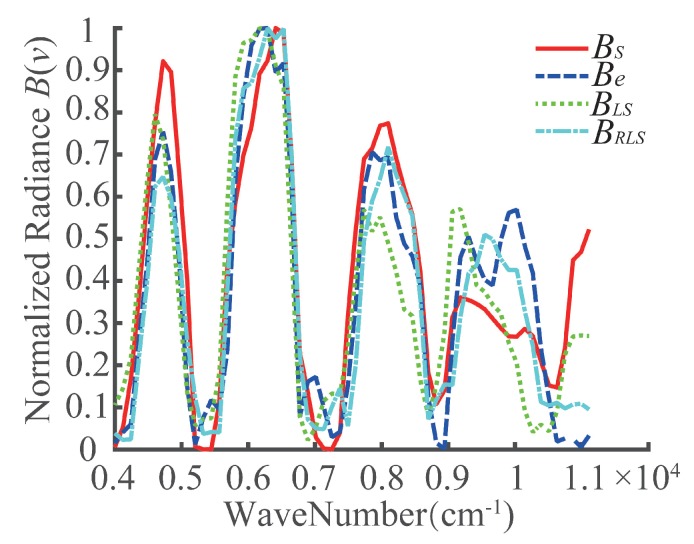
The spectrum of the target *P*.

In Table 3, error denotes the design values of the parameters. The OPD of the interference image was corrected by the calculated registration error, the interferogram of the target point *P* was extracted from the image sequence, and its spectrum was restored. The recovered spectrum of the target *P* is shown in Figure 11, where the ideal spectrum was measured synchronously by an analytical spectra device (ASD) on the ground. In the figure, the red solid line represents the ASD spectrum and labeled with $B_{S}$, the blue dashed line represents the error spectrum labeled with $B_{e}$, the green dotted line represents the spectrum corrected by the LS method and labeled using the symbol $B_{LS}$, and the cyan dot-dashed line represents the spectrum correctedby the RLS method and labeled with the symbol $B_{RLS}$. The figure shows that the positions of the peaks in the curve of $B_{RLS}$ are nearer to $B_{S}$ than the others, and the variation trends of the spectral shape of $B_{RLS}$ are the closest to $B_{S}$, especially near the wave numbers 6000 and 8000 cm${}^{-1}$. The evaluation factor SA is provided in Table 3. From the table, the SA of the RLS method is the lowest. However, the recovered spectrum still does not match the ASD spectrum very well because other errors of the extracted interferogram are present, such as positioning errors of the homonymic point locations in the image series due to unstable platform movement and the interpolation errors of interference information.

## 7. Conclusions

In this study, we analyzed the importance of the correction of the registration error between the detector and the interferogram based on the TSMFTIS imaging principle, and we proposed a two-step correction method for the registration error. The method automatically selects points for parabolic fitting to obtain the zero OPD positions in the interference-modulated image and then calculates the threshold value for the RLS method to obtain an accurate and robust corrected value of the registration error. The effectiveness of the proposed method was verified by simulation. Finally, the correction method was applied to an experimental image sequence, demonstrating that the method provides improved spectral recovery accuracy.

## Figures and Tables

**Table 1:** The parameters of linear fitting of the registration error between the detector and the interferogram.

Group		1		2		3	
Parameter		k	t	k	t	k	t
Preset Value		0	38	−0.01	40.5	−0.02	43
	**(least−squares)LS**	−0.0020	38.0559	−0.0124	40.5949	−0.0122	40.2298
**Fitting**	**(robust−least−squares)RLS**	−1.7$\times 10^{-6}$	38.0011	−0.0100	40.4825	−0.0200	43.0163
**Method**	**(total−least−squares)TLS**	−0.0020	38.2493	−0.0132	40.8506	−0.0130	40.4842
	**(robust−total−least−squares)RTLS**	−1.7$\times 10^{-6}$	38.0011	−0.0100	40.4825	−0.0200	43.0163

**Table 2:** Spectral angle (SA) caused by the registration error between the detector and the interferogram.

Target	$A_{1}$			$A_{2}$		
Case	1	2	3	1	2	3
**Error**	0	0.0736	0.1470	0	0.2746	0.2094
**LS**	0.2197	0.2644	0.3814	0.3168	0.2861	0.3057
**RLS**	0.0007	0.0205	0.0033	0.0005	0.0235	0.0099

**Table 3:** The fitting parameters and SA of different correction methods for the registration error.

Parameter Name		Error	LS	RLS
**Linear Fitting**	***k***	0	−0.0125	−0.0143
**Parameters**	***t***	38	39.0844	39.3588
**Evaluate Factor**	**SA**	0.3306	0.3415	0.3031

## Abbreviations

The following abbreviations are used in this manuscript:

TSMFTIS	temporally-spatially modulated Fourier transform imaging spectrometer
OPD	optical path differences
LASIS	large-aperture static imaging spectrometer
HEIFTS	high etendue imaging Fourier transform spectrometer
CaHyD	Caméra Hyperspectrale de Démonstration
ALISEO	aerospace leap-frog imaging static interferometer for Earth observation
FPA	focal plane array
LS	least-squares
TLS	total-least-squares
RLS	robust-least-squares
RTLS	robust-total-least-squares
SA	spectral angle
ASD	analytical spectra device
